# Comparing the Expressions of Vitamin D Receptor, Cell Proliferation, and Apoptosis in Gastric Mucosa With Gastritis, Intestinal Metaplasia, or Adenocarcinoma Change

**DOI:** 10.3389/fmed.2021.766061

**Published:** 2021-11-22

**Authors:** Li-Wei Chen, Liang-Che Chang, Chung-Ching Hua, Tzu-Chien Cheng, Chin-Chan Lee

**Affiliations:** ^1^Department of Gastroenterology and Hepatology, Chang-Gung Memorial Hospital and University at Keelung, Keelung, Taiwan; ^2^Community Medicine Research Center, Chang-Gung Memorial Hospital and University at Keelung, Keelung, Taiwan; ^3^Department of Pathology, Pathology Chang-Gung Memorial Hospital and University at Keelung, Keelung, Taiwan; ^4^Department of Internal Medicine, Internal Medicine Chang-Gung Memorial Hospital and University at Keelung, Keelung, Taiwan

**Keywords:** adenocarcinoma, stomach neoplasm, cell proliferation, apoptosis, artificial intelligence, Ki67 antigen, keratin 18, receptor calcitriol

## Abstract

**Background:** This study aimed to compare the expression of vitamin D receptor (VDR), cell proliferation, and apoptosis in the gastric mucosa of patients with gastritis, intestinal metaplasia (IM), and adenocarcinoma using artificial intelligence.

**Material and Methods:** This study retrospectively enrolled patients at the Keelung Chang Gung Memorial Hospital from November of 2016 to June, 2017, who were diagnosed with gastric adenocarcinoma. The inclusion criteria were patients' pathologic reports that revealed all compartments of *Helicobacter pylori* infection, gastritis, IM, and adenocarcinoma simultaneously in the same gastric sample. Tissue slides after immunohistochemical (IHC) staining were transformed into digital images using a scanner and counted using computer software (QuPath and ImageJ). IHC staining included PA1-711 antibody for VDR, Ki67 antigen for proliferation, and M30 antibody CK18 for apoptosis.

**Results:** Twenty-nine patients were included in the IHC staining quantitative analysis. The mean age was 69.1 ± 11.3 y/o. Most (25/29, 86.2%) patients had poorly differentiated adenocarcinoma. The mean expression of Ki67 and CK18 increased progressively from gastritis and IM to adenocarcinoma, with statistical significance (*P* < 0.05). VDR expression did not correlate with Ki67 or CK18 expression. Survival time was only correlated with tumor stage (correlation coefficient = −0.423, *P* value < 0.05), but was not correlated with the expression of VDR, Ki67, and CK18.

**Conclusion:** Ki67 expression and CK18 expression progressively increased in the areas of gastritis, IM, and adenocarcinoma. No correlation between VDR expression and Ki67 or CK18 expression was found in this study.

## Introduction

Gastritis and intestinal metaplasia (IM) are common findings in patients with *Helicobacter pylori* (*H. pylori*) infection (1, 2). The prevalence of IM in patients with *H. pylori* infection is 30–40% at the age of 50 years old (1, 2). Gastric epithelial hyperproliferation has been observed in patients with gastritis and IM caused by *H. pylori* infection (3–5). Previous studies have also revealed that IM is associated with an increased risk of gastric cancer (6–9). Detection of IM in gastric adenocarcinoma samples is a common histological finding (9).

Apoptotic cells are rare in the glandular neck region (the generative cell zone) of normal gastric mucosa. With progression of atrophic gastritis, the generative cell zone shifts downward and a relatively large number of apoptotic cells occur (10). *H. pylori* infection induces apoptosis in gastric epithelial cells (10). The effect of *H. pylori* apoptosis could result from molecules produced by *H. pylori* or the host immune/inflammatory response (10). Molecules such as cytotoxin (VacA), lipopolysaccharide, or nitric oxide may directly induce apoptosis (10, 11). Many cytokines produced by type 1 T helper cells, such as TNF-α and IFN-γ, markedly potentiate apoptosis. The balance between cell proliferation and apoptosis is important for carcinogenesis in precancerous lesions, such as IM and *H. pylori* infection (10, 11).

Gut epithelial vitamin D receptor (VDR) signaling appears to play an essential role in controlling mucosal inflammation and thus could be a useful therapeutic target in the management of some gastrointestinal diseases (12). Although 1, 25-Dihydroxyvitamin D [1, 25 (OH)_2_D3] is not produced by the stomach, it affects the immune regulatory responses via the VDR of the stomach (13–15). Vitamin D deficiency has been associated with risk of several cancers including gastric cancer (16). VDR is a superfamily of steroid hormone receptors that act as a transcription factor for a target gene. Moreover, 1, 25 (OH)_2_D3 was reported to be associated with the inhibition of cell cycle progression, induction of cell apoptosis, and differentiation of various types of cancer cells. Hence, 1, 25 (OH)_2_D3 has been reported to inhibit proliferation and anti-tumor effects via VDR (15, 16). VDR was also reported to play an important role in gastric mucosa homeostasis and host protection against *H. pylori* infection (13). VDR could regulate cathelicidin antimicrobial protein (CAMP) and has an antimicrobial activity against *H. pylori* (13, 17). Past studies found an important role of the VDR/CAMP pathway in innate immunity and an anti-inflammatory mechanism of vitamin D. VDR mRNA expression levels were significantly up-regulated in *H. pylori*-infected patients and positively correlated with chronic inflammation scores. There was a significant positive correlation between VDR and CAMP mRNA expression in *H. pylori*-positive gastric mucosa (13). In animal model study using wild-type and VDR knockdown mice to demonstrate that VitD3 inhibits H. pylori infection by enhancing the expression of VDR and CAMP. VDR knockdown mice were more susceptible to H. pylori infection. In cultured mouse primary gastric epithelial cells, VitD3/VDR complex binds to the CAMP promoter region to increase its expression (17).

1, 25 (OH)2D3 binding to VDR could transcriptionally activate the expression of a number of target genes, finally executing the antitumor functions. A lot of genes have been identified as its direct targets, such as p21 (18, 19) and c-Myc (20) which are involved in different signaling pathways during tumor genesis. A previous study has shown that vitamin D suppresses proliferation and stimulates cell cycle arrest in gastric cancer cells but not in immortalized normal gastric cells (12). Vitamin D has increased p21 expression and decreased cyclin-dependent kinase 2 (CDK2) expression via VDR route (12).

The hypothesis of this study was that patients infected with *H. pylori* might have gastritis, gastric IM, and gastric adenocarcinoma. VDR expression in the stomach may differ in the areas of gastritis, IM, and gastric adenocarcinoma. When compared with the area of gastritis, the pathologic presentation of cell proliferation and apoptosis may be different in the areas of IM and adenocarcinoma. A progressive increase or decrease in cell proliferation or apoptosis may be detected in the areas of gastritis, IM, and gastric cancer, respectively.

The current study aimed to evaluate VDR expression, cell proliferation, and apoptosis in gastric adenocarcinoma samples using digital quantitative immunohistochemistry (IHC) statin analyses. In this study we aimed to:

1. Compare the Expression of VDR, Cell Proliferation (by Ki67 Statin), and Apoptosis (by CK18 Staining) in three Different Parts, Including Gastritis (non-Dysplasia or Tumor), IM (Premalignant Area), and Gastric Adenocarcinoma (Malignant Site) by Artificial Intelligence (AI) Using Computer Software to Prevent Manual or Inter-Observer Bias for IHC Score Counting;
2. Analyze the Association Between VDR Expression, Cell Proliferation, and Apoptosis;
3. Elucidate Whether High VDR Expression Is Associated With Higher or Lower Cell Proliferation and Apoptosis;
4. Analyze the Correlation of Survival Time With Tumor Stage and three IHC Stain Expressions.

## Materials and Methods

### Subjects and Tissue Samples

This study retrospectively enrolled patients who were diagnosed with gastric adenocarcinoma at the Keelung Chang Gung Memorial Hospital (KCGMH) from November of 2016 to June 2017. Gastric samples that were confirmed as malignant after pathologic examination were stored in the tissue bank of KCGMH. For a patient with both endoscopic biopsy and surgical resection specimens, samples from surgical resection were used for IHC staining expression analyses in this study. For patients without an operation for gastric malignancy, endoscopic biopsy samples were used for IHC analyses. The inclusion criteria were patients' pathologic reports that revealed all compartments of *H. pylori* infection, gastritis, IM, and adenocarcinoma simultaneously in the same gastric sample. The exclusion criteria were incomplete records of demographic data, tumor stage, and clinical course.

Patient demographics, tumor location in the stomach, tumor stage, and survival time were recorded. No chemotherapy, radiotherapy, or other therapies were performed in these patients before endoscopic biopsy or surgical resection. Two pathologists (Dr. Chang LC and Dr. Cheng TC) provided all the equipment and histological examinations. Every specimen was reviewed by an experienced pathologist (Dr. Chang LC) under microscopic examination to localize the areas of gastritis, IM, and adenocarcinoma. Commercial kits for IHC staining and publicly available software applications for digital image creation and quantitative analysis were used. This study was approved by the Ethics Committee of the Chang Gung Memorial Hospital (IRB No 103-7463A3, 105-4426C).

### Histology and IHC Stain for *H. pylori* Detection and IM

Histology (hematoxylin and eosin) and IHC *H. pylori* antibody staining (polyclone, Zytomed Systems GmbH, Berlin, Germany) were performed to confirm *H. pylori* infection. Histological sections of all specimens were routinely examined to determine gastritis, IM, and malignancy. Because the gastric mucosa adjacent to malignancy is always infiltrated by inflammatory cell, a diagnosis of gastritis is made for this non-tumor, non-IM mucosa by pathologists. IM was detected based on the morphological features in the stomach observed by H&E and Alcian blue staining (6, 21).

### Digital Quantitative Analyses for IHC Images

Tissue sections were cut from the tissue blocks at 4 μm and stained with H&E and IHC. The slides were scanned at 400 × magnification using a Hamamatsu Nanozoomer S360 scanner and NDP image (Hamamatsu, Japan). Digital data analysis was performed using computer software to prevent manual or inter-observer bias for IHC score counting. The computer softwares ImmunoRatio (ImageJ plugin) (22) and QuPath (23) were applied for digital slide bioimage analyses (24–26). The application of ImmunoRatio calculates the percentage of positively stained nuclear area (labeling index) by using a color deconvolution algorithm for separating the staining components (diaminobenzidine and hematoxylin) and adaptive thresholding for nuclear area segmentation (25). Every digital image was quantitatively analyzed in three adjacent areas: gastritis, IM, and adenocarcinoma.

### Ki-67 Antigen for Epithelial Cell Proliferation

Antigen Ki-67 IHC staining was performed using a DAKO autostain agent (Cytomation, Carpinteria, CA, USA). The REAL EnVision Detection System, Peroxidase/diaminobenzidine (DAB) (K5007, DAKO), was used to visualize the staining.

### PA1-711 Antibody for VDR

After appropriate blocking and management of gastric tissue, VDR staining was performed using an anti-VDR polyclonal antibody (PA1-711, Thermo Scientific, Fremont, CA, USA).

### M30 CytoDEATH Antibody for Detecting Apoptosis

Mouse monoclonal antibody (Clone M30, mouse IgG2b) was used to detect apoptosis in epithelial cells (caspase cleavage product of cytokeratin 18, CK18). Apoptosis was detected by applying the M30-antibody to fixed samples, and then secondary detection systems were used for IHC staining. The M30 CytoDEATH antibody (Roche Diagnostics GmbH, Mannheim, Germany) binds to a caspase-cleaved, formalin-resistant epitope of the CK 18 cytoskeletal protein (27).

### Gastric Cancer Stage

Gastric cancer staging was performed according to the American Joint Committee on Cancer (AJCC, 7th edition) (28).

### Statistical Analysis

Continuous data are expressed as mean ± standard deviation (SD). A two-sample *t*-test was used to compare the mean values. Categorical data were analyzed using chi-square and Fisher exact tests. One-way analysis of variance (ANOVA) was used to compare the mean values of multiple samples. The Scheffe method was applied for *post-hoc* analysis. All statistical tests were two-tailed. Differences were considered statistically significant at *p* < 0.05. Statistical analyses were performed using the Statistical Package for the Social Sciences (version 18.0) for Windows (PASW, Chicago, IL, USA).

## Results

**Figure 1:**
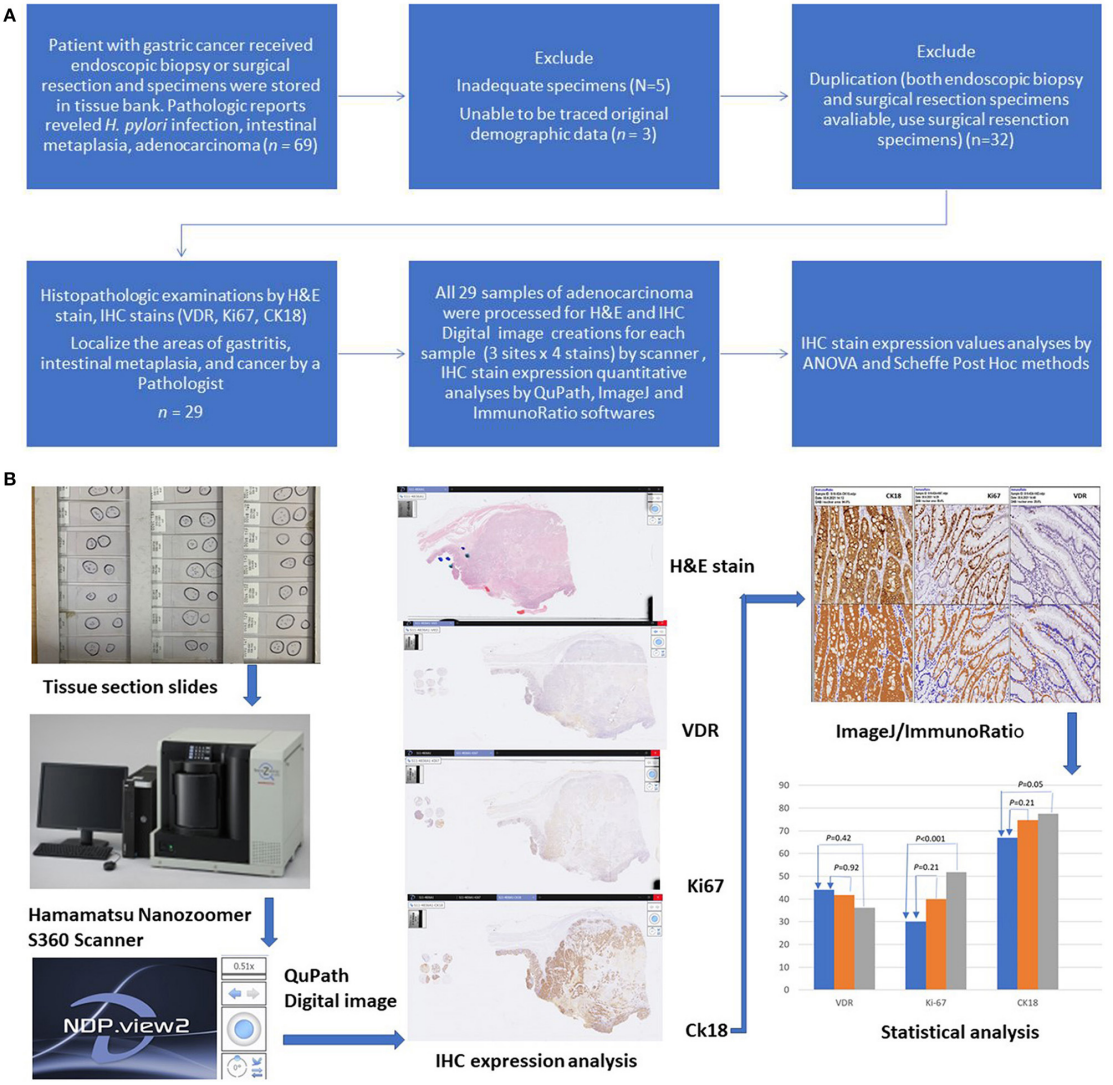
**(A)** Study flow diagram. All 29 samples of adenocarcinoma were processed for H&E and IHC stain. **(B)** Graphic flow chart.

Initially, 69 gastric tissue samples were collected from the tissue bank of KCGMH. Among these 69 samples, 32 originated from the same patients (from endoscopic biopsy and surgical resection). Five samples were inadequate for IHC staining examination. We were unable to trace the origins of three samples. Finally, 29 patients (24 surgical resections and 5 endoscopic biopsies) were included in the IHC staining analysis (Figure 1, Study flow). IHC stains of PAI 711 antibody (VDR), Ki67 antigen (cell proliferation), and M30 antibody CK18 (cell apoptosis) were performed for all samples from these 29 patients.

The demographic and clinical characteristics of the patients are listed in Table 1. The mean age was 69.1 ± 11.3 y/o. Most (25/29, 86.2%) patients had poorly differentiated adenocarcinoma. The tumors were located in the gastric antrum (22/29, 75.9%), body (6/29, 20.7%), and cardiac (1/29, 3.4%). Tumor stages at diagnosis were stage I (13/29, 44.8%), stage II (2/29, 6.9%), stage III (6/29, 20.7%), and stage IV (8/29, 27.6%).

**Table 1:** Demography and clinical characters.

Age (y/o)	69.1 ± 11.3
Gender (F/M)	12/17
Adenocarcinoma type (tubular/other)	(27/2)
Tumor location (antrum/body/cardiac)	22/6/1
Differentiation (poor/moderate/well)	25/4/0
Operation (with/without) after diagnosis	24/5
Operation type (BI/BII/Total gastrectomy)	14/8/2
Tumor stage (I/II/III/IV)	13/2/6/8
Chemotherapy (with/without) after diagnosis	15/14
Mean survival days (stage I/II/III/IV)	2631/1472/920/274

*IM, intestinal metaplasia; BI, Billroth's I subtotal gastrectomy; BII, Billroth's II subtotal gastrectomy; Total, total gastrectomy*.

**Figure 2:**
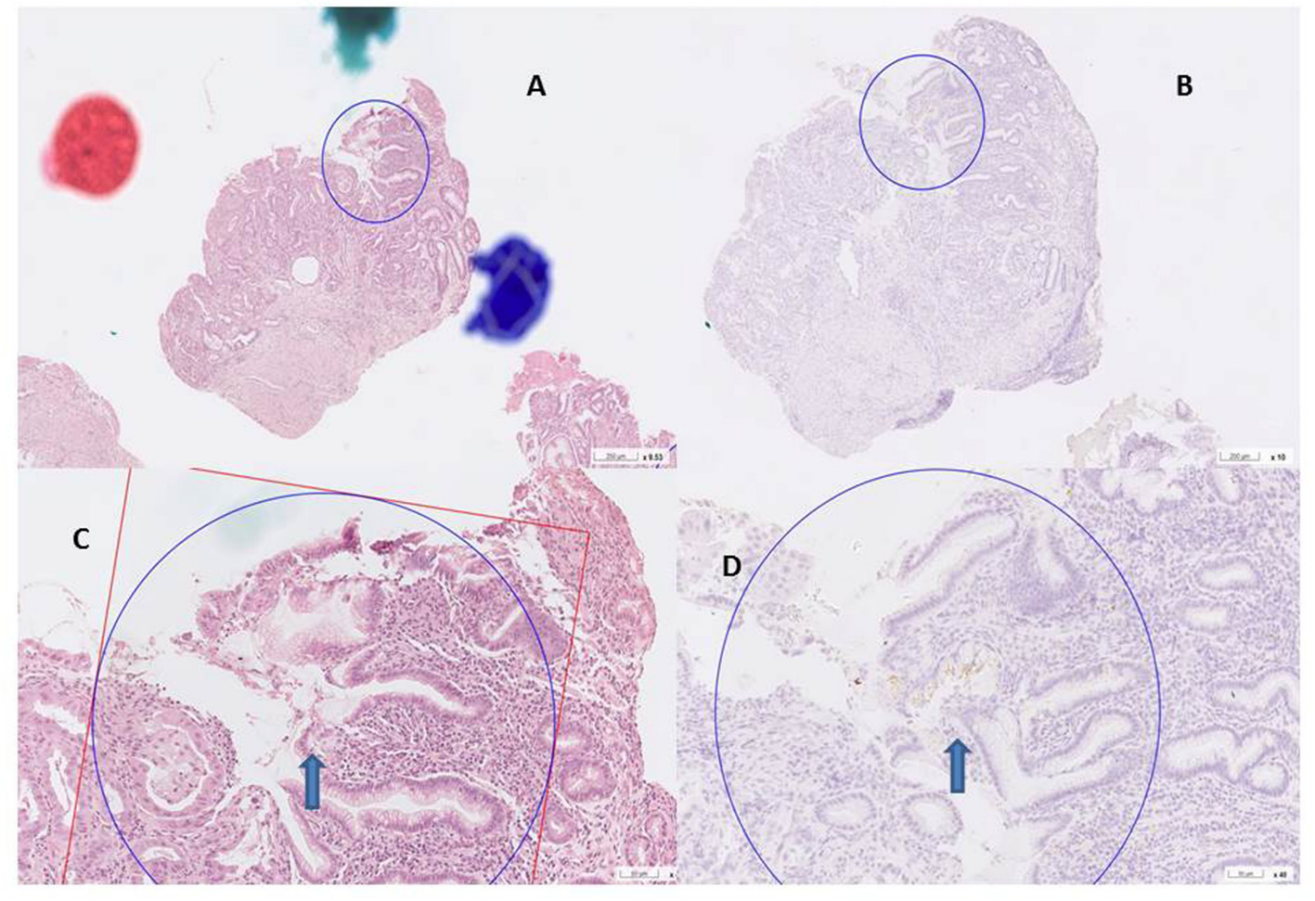
*H. pylori* infection H&E and antibody stain. **(A,B)** H& E stain, curved bacilli within the mucus of epithelim (arrow). **(C,D)** anti *H. pylori* antibody stain (arrow).

**Figure 3:**
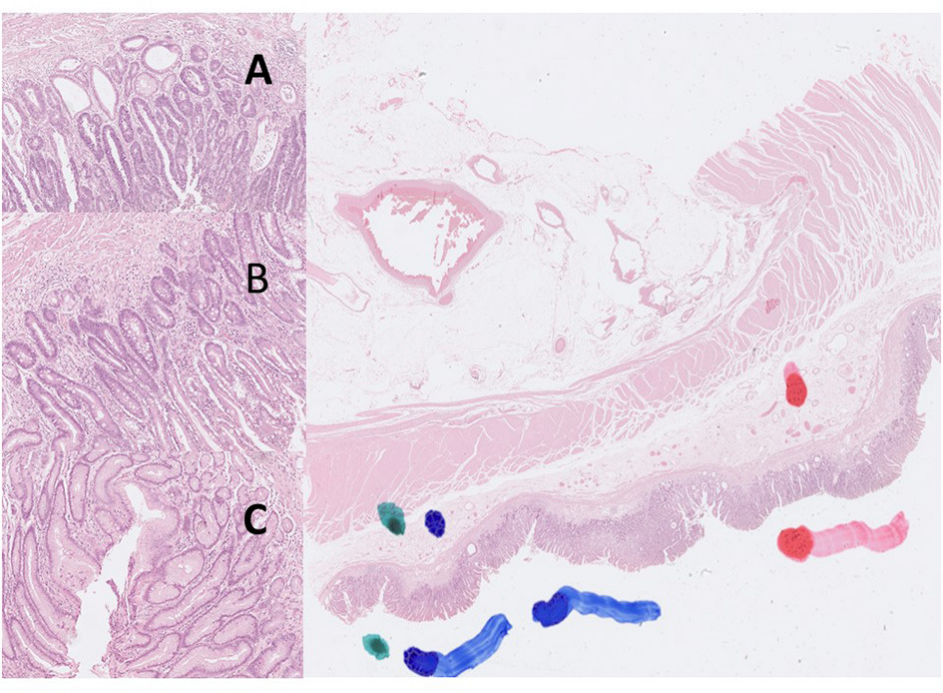
Digital pictures of H&E stain created by Hamamatsu Nanozoomer S360 scanner and QuPath software (40X). The color markers were made by a pathologist to localize the targeted areas (green color: gastritis; blue color: intestinal metaplasia; red color: gastric adenocarcinoma). **(A)** adenocarcinoma, **(B)** intestinal metaplasia, **(C)** gastritis.

**Figure 4:**
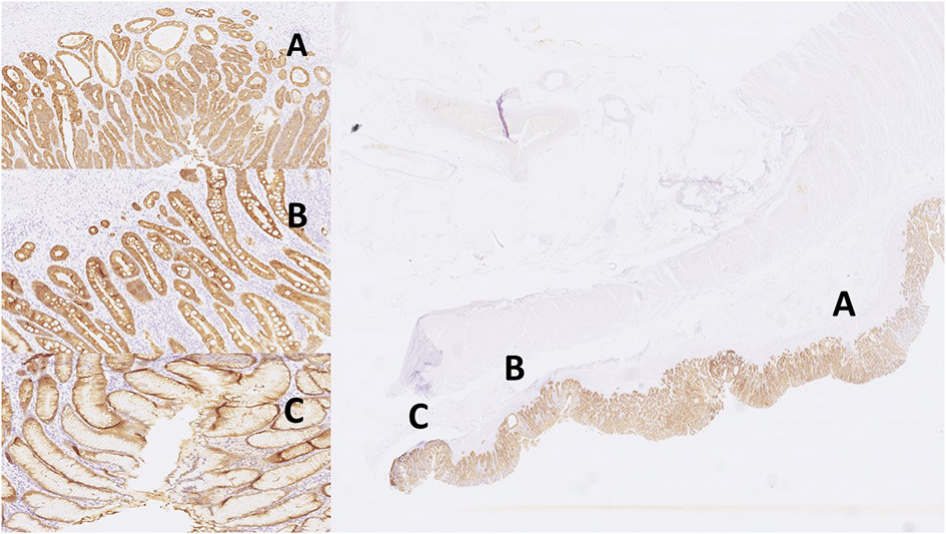
Images of IHC expression by CK18 stain in different sites. Right: total; left top: gastritis; left middle: IM; left bottom: adenocarcinoma. **(A)** adenocarcinoma, **(B)** intestinal metaplasia, **(C)** gastritis.

**Figure 5:**
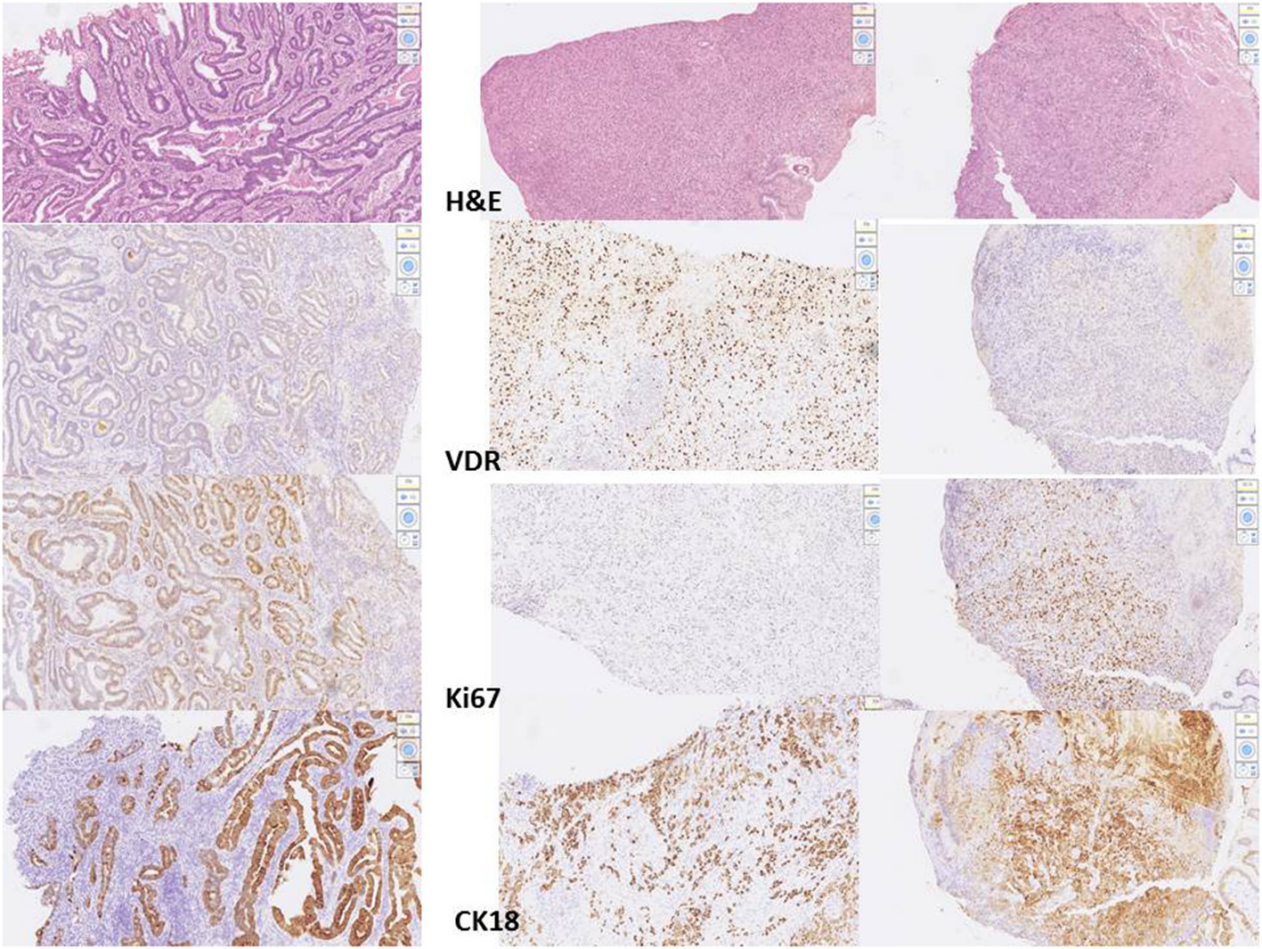
Digital images for different cell type of adenocarcinoma from 3 patients. Left: moderate differentiated adenocarcinoma, stage III. Middle: poor differentiated adenocarcinoma, stage I. Right: poor differentiated adenocarcinoma, stage III. From the top to the bottom: H&E stain, VDR, Ki67, CK18 stain processed by scanner and QuPath software.

**Figure 6:**
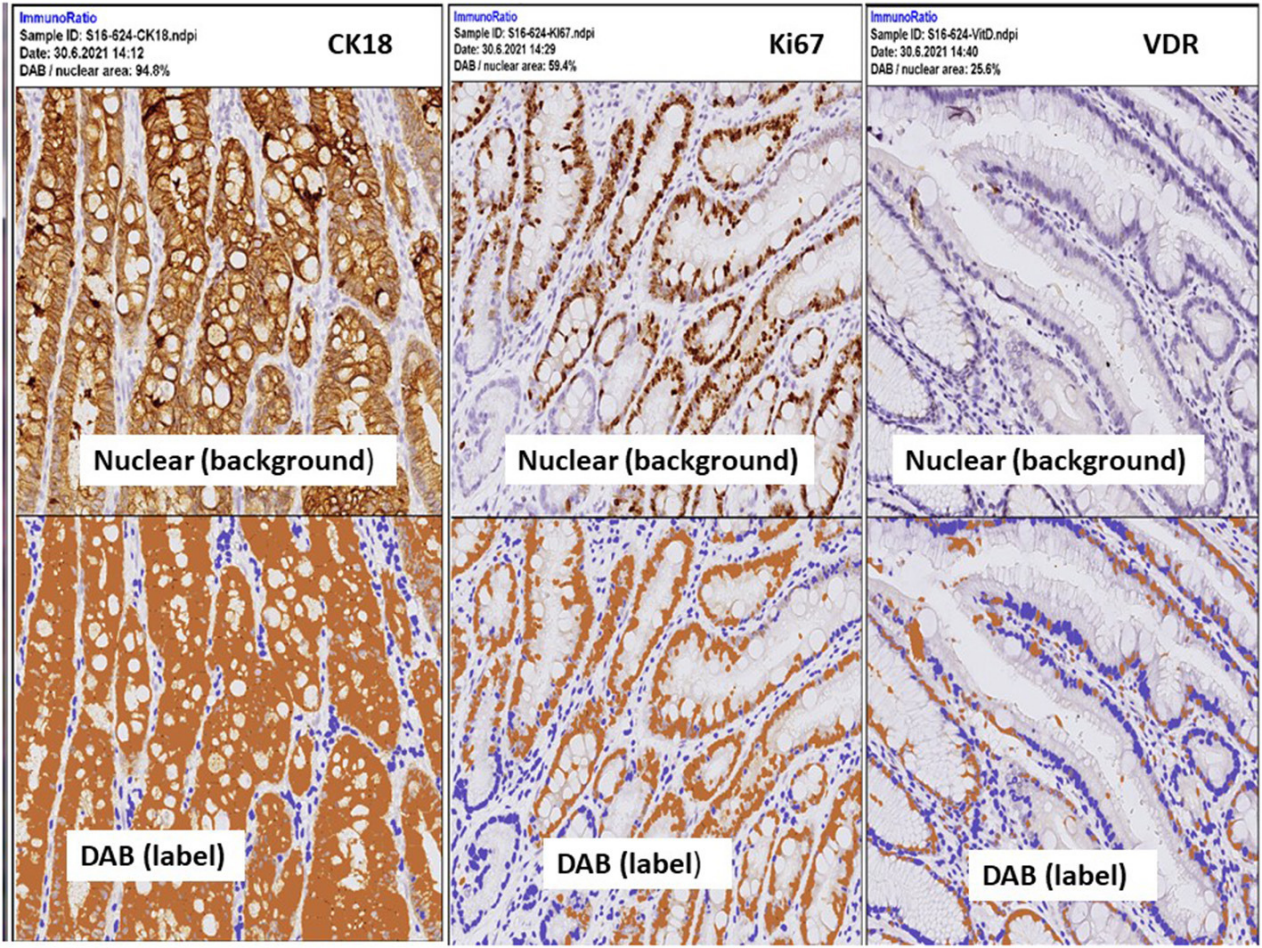
IHC expressions by ImmunoRatio (ImageJ plugin software) processed from one patient with stage I, poor differentiated type gastric adenocarcinoma. Upper panel: the original hematoxylin image (total cell). Lower panel: “DAB” indicates labeled cells, a pseudo-colored image showing the segmented staining components. Labeling index is calculated by the percentage of DAB (positively stained cells) divided the total cell. Left: 94.8% by CK18, middle: 59.4% by Ki67, right: 25.8% by VDR.

Digital images of *H. pylori* infection (H&E and antibody stain, Figure 2). Figures 3, 4 reveals digital images of H&E and CK18 stain in different sites (total, gastritis, IM, and adenocarcinoma). Digital analyses of IHC expression using ImageJ and ImmunoRatio software are presented in Figure 5. Figure 6 reveals different cell types (moderate differentiation and well-differentiation) gastric adenocarcinoma and IHC (VDR, Ki67 and CK18) stain.

The expression levels of PAI 711 antibody (VDR), Ki67 antigen (cell proliferation), and M30 antibody CK18 (cell apoptosis) among gastritis, IM, and adenocarcinoma are listed in Table 2. The mean expression of Ki67 and CK18 increased significantly (*P* < 0.05) from gastritis, IM to adenocarcinoma. However, mean values of VDR expression were no statistical difference among gastritis, IM, and adenocarcinoma by ANOVA (*P* = 0.404).

**Table 2:** The mean expressions of VDR, Ki67, and CK18 among gastritis, IM and cancer.

	**Mean expression (%) in targeted areas**			
**IHC stain**	**Gastritis**	**IM**	**Cancer**	***P*-value**
VDR	44.1 ± 26.4	41.8 ± 16.9	36.1 ± 19.7	0.404
Ki67	30.1 ± 18.4	39.9 ± 18.5	51.8 ± 21.8	<0.001
CK18	66.9 ± 19.8	74.7 ± 15.0	77.6 ± 14.7	0.047

*IHC stain, immunohistochemical stain; Gastritis, inflammation but relatively normal gastric mucosa adjacent to malignancy; IM, intestinal metaplasia adjacent to malignancy; Cancer, gastric adenocarcinoma; VDR, PA1-711 for vitamin D receptor; Ki67, antigen Ki-67 stain for epithelial cell proliferation; CK18, cytokeratin 18, M30 CytoDEATH antibody for detecting apoptosis; P-value, ANOVA analysis*.

**Figure 7:**
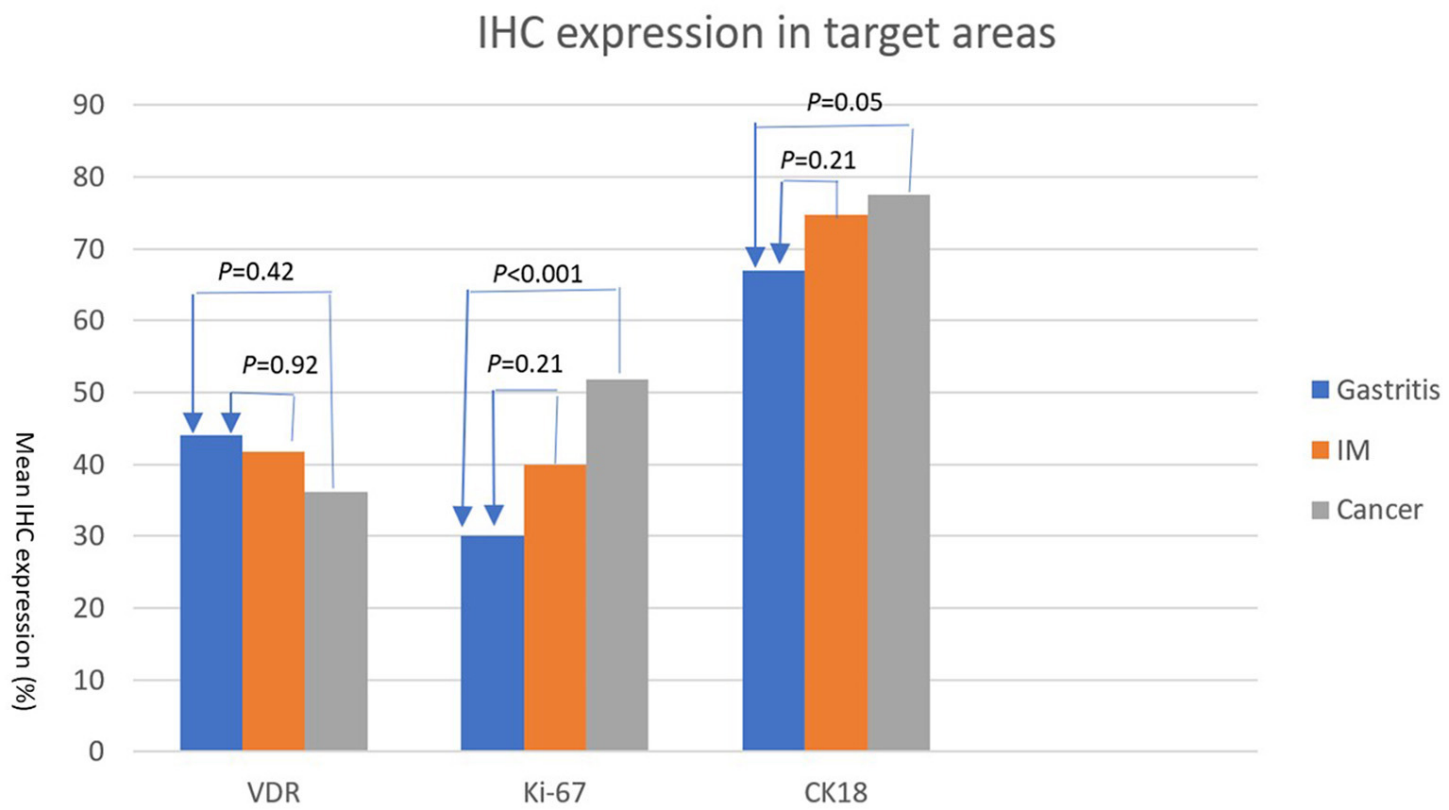
IHC expressions (%) (Y axis) of VDR, Ki67, and CK18 (X axis) in areas of gastritis, IM, and cancer. The expression of each IHC stain in IM (orange bar) and cancer (gray bar) is compared with the expression in gastritis (blue bar). *P*-values are presented at the top of each comparison (Scheffe *post-hoc* analysis).

Table 3 shows the Scheffe *post-hoc* analysis. The main location for a different expression of Ki67 and CK18 were between gastritis and cancer. The expression of Ki67 and CK18 was similar between the IM (premalignancy) and adenocarcinoma (malignancy) sites (Figure 7).

**Table 3:** Scheffe *post hoc* analysis among different stains and locations.

**Stain**	**VDR**		**Ki67**		**CK18**	
**Location**	**Gastritis**	**IM**	**Gastritis**	**IM**	**Gastritis**	**IM**
**Adenocarcinoma+**						
Coefficient	−7.362	−5.103	21.128	11.931	10.638	2.851
*P*-value	0.421	0.659	0.000	0.069	0.057	0.809

*+ Post hoc compare the expression of IHC between adenocarcinoma and gastritis or intestinal metaplasia*.

When a correlation analysis between VDR and Ki67 or CK18 was performed, VDR expression was not correlated with Ki67 or CK18 expression in gastritis, IM and adenocarcinoma site (Table 4).

**Table 4:** Correlation the expressions between VDR and Ki67 or CK18 in different sites.

**Area**	**Compared factor**	**Correlation**	***P*-value**
**referred factor**		**coefficient**	
**Gastritis**			
VDR	Ki67	0.142	0.462
	CK18	−0.121	0.531
**IM**			
VDR	Ki67	0.171	0.375
	CK18	−0.302	0.111
**Cancer**			
VDR	Ki67	0.066	0.736
	CK18	0.103	0.597

Compare the VDR expression in gastritis, IM, and adenocarcinoma between male and female patients. There was no statistical difference of mean VDR expression between male and female patients in the gastritis (42.2 ± 25.0 vs. 48.9 ± 27.7, *P* = 0.58), IM (41.8 ± 17.3 vs. 41.7 ± 14.7, *P* = 0.73), and adenocarcinoma (36.6 ± 18.8 vs. 36.6 ± 18.5, *P* = 0.98).

Survival time was only correlated with tumor stage (correlation coefficient = −0.423, *P-*value <0.05), but was not correlated with the expression of VDR, Ki67, and CK18.

## Discussion

VDR could regulate cathelicidin antimicrobial protein (CAMP) and has an antimicrobial activity against *H. pylori* (13, 17). Past studies found an important role of the VDR/CAMP pathway in innate immunity and an anti-inflammatory mechanism of vitamin D (13, 17). VDR mRNA expression levels were significantly up-regulated in *H. pylori*-infected patients and positively correlated with chronic inflammation scores. There was a significant positive correlation between VDR and CAMP mRNA expression in *H. pylori*-positive gastric mucosa (13). Previous studies have shown that VDR expression may control the expression of cell proliferation and apoptosis (14–16, 29–31). Wen et al. (32) demonstrated that VDR was lowest in cancer tissues (positive rate = 57.61%), compared to premalignant (73.64%) and normal tissues (82.61%), with a statistically significant difference (*P* = 0.001). A decline in VDR expression was observed in normal, premalignant, and malignant gastric tissues, especially in poorly differentiated tissues. In our study, there was no statistically difference of VDR expression (44.1%, 41.8%, 36.1% in gastritis, IM, and malignant tissue, respectively; *P* = 0.404) was detected. The main reason for the difference in results between Wen et al. (32) and our study may be due to the different methods for VDR expression evaluation. In Wen et al. study, VDR expression was presented as a positive rate (PR), which was similar to qualitative analysis (positive or negative). Moreover, some gastritis or IM tissues were collected from patients without gastric cancer. In our study, quantitative digital analyses were performed to prevent intra-or inter-observer bios. All three parts (gastritis, IM, and cancer) were adjacent and came from the same patient. Because the major type of gastric adenocarcinoma was poorly differentiated in this study, similar VDR expression in the mucosa adjacent to the tumor may be found in patients with poorly differentiated gastric adenocarcinoma.

The relationship between serum vitamin D deficiency and gastric cancer development remains under debate (13–15, 33, 34). Because 1, 25 (OH)_2_D3 is not produced by the stomach, 1, 25 (OH)_2_D3 affects the immune regulatory responses via the VDR of the stomach (12–14). However, no serum vitamin D level was recorded in this study because checking the vitamin D level was not a regular guideline for gastric adenocarcinoma treatment. To understand the relationship between serum vitamin D level and gastric VDR expression, it is necessary to include more patients with serum vitamin D and VDR expression in the gastric mucosa by endoscopic biopsy.

It is common to disclose increased proliferation and decreased apoptosis in malignant cells (10). Our hypothesis was an increased Ki67 (cell proliferation) and a deceased CK18 (apoptosis) in cancer, but the results of our study revealed that both Ki67 and CK18 expression increased in IM and malignant tissues. Apoptosis is defined by characteristic changes in nuclear morphology, including chromatin condensation and fragmentation, overall cell shrinkage, and blebbing of the plasma membrane. CK18 expression was high (mean 77.6%) in gastric adenocarcinoma in the current study. The expression of CK18 was also high in the non-tumor gastritis mucosa in the current study (mean 66.9%).

Chronic inflammation and IM are associated with increased apoptosis but primarily occur at the mucosal surface and not in the deeper layers (27). In the current study, the mucosa of gastritis was adjacent to the IM and the malignant areas. Chronic inflammation may induce high CK18 expression (35). Previous studies on apoptosis in gastric adenocarcinoma mostly used cell lines and the counting score for apoptosis was variable (10, 35). CK18 expression in the mucosa of gastritis adjacent to IM and gastric adenocarcinoma (especially poor differentiation type) may be high in nature. An imbalance between cell proliferation and apoptosis may be the reason and mechanism of carcinogenesis (10, 17). Wagner et al. (29) found decreased apoptotic activity following increased proliferation in chronic *H. pylori* infection; they assumed that increased proliferation might play a role in carcinogenesis.

In normal gastric mucosa, apoptotic cells are rare and in the generative cell zone near glandular neck region. When the sequential change of atrophic gastritis, IM, and dysplasia, the number of apoptotic cells increase (10). Numerous molecules produced by *H. pylori* including cytotoxin (VacA), lipopolysaccharide, monochloramine, and nitric oxide may directly induce apoptosis. Moreover, *H. pylori*-stimulated host inflammatory/immune responses lead to release of a large amount of cytokines. Cytokines produced by type 1 T helper cells, such as TNF-alpha and IFN-gamma, markedly potentiate apoptosis (10, 36).

This study applied artificial intelligence (AI) methods, including a high-resolution scanner (Hamamatsu Nanozoomer S360 scanner) for digital image creation, QuPath, and ImageJ/ImmunoRatio software for IHC scoring. This software could be downloaded freely from specific webs. The strength of AI use is rapid and avoids intra- or inter-observer bios. AI for IHC score counting is easier than traditional manual counting. The preparing of samples in this study were paraffin-embedded tissue sections from tissue bank. Paraffin-embedded tissues are frequently used for pathological examinations, included IHC analyses. Patients who get *H. pylori* infection may develop the conditions of gastritis, IM and gastric adenocarcinoma, which conditions could be detected in one slicing slide from the surgical or biopsy specimens. IHC examination could evaluate the expressions of VDR, Ki67 and CK18 with precise locations in one slide simultaneously. However, there are some limitations to use AI for quantitative IHC tests like our study. First, the precisely targeted areas for scanning and scoring still depend on the pathologists in our study. Second, reports issued the correlation between IHC quantitative analyses and gene expressions in this field of *H. pylori* related gastric adenocarcinoma are rarely now. Third, the application of ImageJ/ImmunoRatio software for other different IHC examinations may require further studies. Hence, further studies are needed for validation and clarification the correlation between digital IHC quantitative tests and gene expression examination.

Conclusively, cell proliferation by Ki67 expression and apoptosis by CK18 expression progressively increased in the areas of gastritis, IM, and adenocarcinoma. Ki67 expression positively correlated with CK18 expression in gastritis. No association between VDR expression and Ki67 or CK18 expression was found in this study. Survival time was only correlated with tumor stage, but was not correlated with the expression of VDR, Ki67, and CK18.

## Data Availability Statement

The original contributions presented in the study are included in the article, further inquiries can be directed to the corresponding author.

## Ethics Statement

The Institutional Review Board of the Chang-Gung Memorial Hospital approved this research (IRB No. 103-7463A3, 105-4426C). All participants agreed to the study conditions and provided informed consent before the enrollment in this study.

## Author Contributions

L-WC, L-CC, and C-CH contributed to conception and design of the study. L-WC organized the database, wrote the first draft of the manuscript, and got the grant. L-WC, C-CH, and C-CL performed the statistical analysis. L-WC and L-CC wrote sections of the manuscript. L-CC and T-CC provided all the equipment and histological examinations. All authors contributed to manuscript revision, read, and approved the submitted version.

## Funding

This study was supported by the Chang Gung Medical Foundation and Keelung Chang Gung Memorial Hospital Tissue Bank (CRRPG2H0052).

## Conflict of Interest

The authors declare that the research was conducted in the absence of any commercial or financial relationships that could be construed as a potential conflict of interest.

## Publisher's Note

All claims expressed in this article are solely those of the authors and do not necessarily represent those of their affiliated organizations, or those of the publisher, the editors and the reviewers. Any product that may be evaluated in this article, or claim that may be made by its manufacturer, is not guaranteed or endorsed by the publisher.
